# Heat Treatment of AZ91 Magnesium Alloy Coated with an Al_2_O_3_ Thin Film with Fluidized Bed Technology

**DOI:** 10.3390/ma12020216

**Published:** 2019-01-10

**Authors:** Gabriele Baiocco, Gianluca Rubino, Nadia Ucciardello

**Affiliations:** 1 Dipartimento di Ingegneria Industriale e dell’Informazione e di Economia, University of L’Aquila, Via G. Gronchi 18, 67100 L’Aquila, Italy; 2 Università della Tuscia, DEIM, Largo dell’Università, 01100 Viterbo, Italy; gianluca.rubino@unitus.it; 3 Dipartimento di Ingegneria dell’Impresa “Mario Lucertini”, University of Rome of Tor Vergata, Via del Politecnico 1, 00133 Rome, Italy; nadia.ucciardello@uniroma2.it

**Keywords:** fluidized bed (FB), magnesium alloy, alumina coating, heat treatment, functional coating

## Abstract

Fluidized bed technology is a methodology widely known in the manufacturing environment for surface treatment of metals. Within the field of surface coating, it has already been exploited for the coating of a magnesium alloy creating a compact layer of Al_2_O_3_. The result was an improvement in mechanical and tribological properties, along with improved corrosion resistance. In this context, the work proposed is addressed towards the evaluation of the effects of thermal post-treatment on the alumina coating produced by means of the fluidized-bed technology. To analyse the effects of heat treatments the morphology, composition and hardness of the samples were investigated along with adhesion and wear resistance of the alumina film. The results obtained show how the temperature affects the surface morphology and promotes the diffusion of magnesium towards the alumina superficial layer. The mechanisms triggered by heat treatments increase the adhesion of the surface film obtained in the deposition process, improving its mechanical and tribological properties.

## 1. Introduction

The application of magnesium and magnesium alloys in engineering fields has grown rapidly in the last years. The low density and the high specific strength [1,2] make magnesium alloys attractive for several applications. In particular, automotive and aerospace fields exploit these materials as they lead to the reduction of fuel consumption and CO_2_ emission [3,4,5]. Properties such good castability, good ductility, high dimensional stability, good biodegradability, and biocompatibility make magnesium exploitable in a wide range of domains [6,7,8]. The main problems affecting these materials are represented by the low corrosion resistance, caused by the high chemical reactivity of magnesium [9,10], and low wear resistance [11,12,13]. In particular, sliding motion has been proved to be a serious problem for magnesium alloys [14]. Furthermore, the chemical reactivity represents a limit also for the realization of treatment aimed at the improvement of its features. Chemical coating methodologies and conversion coatings require the use of chemicals that can trigger violent reactions in magnesium when they are brought into contact.

The fluidized-bed (FB) is a well-known technique for manufacturing systems. It allows several environmentally-friendly treatments of specimens with complex geometries using basic equipment. Many processes may be performed by FB. Among them, the powder coating processes of ferrous and non-ferrous alloys [15] are of great interest, as they may enhance tribological and mechanical behaviour improving wear and corrosion resistance [16]. 

The FB process involves the formation of a surface layer of a material characterized by a high hardness, such as metals or ceramics. During the deposition process the powder of the coating material, whose dimensions are macrometric, impacts on the substrate to be covered. These particles are embedded on the soft surface of the substrate gradually accumulating and generating a layer. During the process, two kind of impacts occur: sliding impacts and the rolling impacts. While the former is characterized by high impact speed and angle the latter feature low impact speed and angle. Anyway, both cause the release of alumina powder fragments that get stuck in the sample surface producing the coating. The thickness of the superficial layer as a function of the processing time features an asymptote caused by the embedding phenomenon. Additionally, the impacts of the particles cause both a compressive residual stress in the substrate and an increased hardening of the external layer of the specimen [17,18]. Among the most suitable materials for the production of functional coatings, ceramic materials are one of the most reliable candidates. In particular, Al_2_O_3_ has been proved to provide excellent wear and corrosion resistance. [19,20]. In the context of the magnesium alloys, this material was exploited for the creation of a protective layer by means of the FB deposition process, obtaining an increase in the tribological and corrosion resistance properties of the substrate [21]. However, heat treatments carried out following deposition processes could affect the properties of the coated sample. A thermal treatment was carried out on a thermal-sprayed coating of Y_2_O_3_ stabilized ZrO_2_ and Al_2_O_3_ powder on René 95 super alloy [22]. The experiment was performed at 1300 °C for 5 h in an argon atmosphere and resulted in an enhancement of the thermal conductivity. A two-coating process was coated (Ni-P and Ni-P-Al_2_O_3_) by means of an electroless process on an Al-Si sample [23]. At a later stage heat treatments were executed at 400–550 °C for 1–8 h under an argon protective atmosphere for the wear resistance improvement evaluation. Heat treatments were produced on 316L stainless steel produced by means of a selective laser melting process and the addition of different volume fraction of TiB_2_ powder [24]. Differently from the previous works, following a heating at 1150 °C two different kind of cooling processes were achieved. The aim of the study was to assess the effect of a secondary annealing treatment on the composite properties. The fluidized bed method was exploited for the production of an Al_2_O_3_ on an aluminium alloy substrate [25]. The following thermal treatment performed was designed in function of heating temperature, up to 600 °C, and process duration. It has been proven to be effective in the enhancement of mechanical and tribological properties of the substrate. In this paper, heat treatments on samples of the magnesium alloy AZ91, in which mechanical properties are reported [26], following a deposition process of Al_2_O_3_ by means of the fluid-bed technology are proposed. Differently from the previous works [22,23,24] and in similarity with [25], the experiment was executed in an air atmosphere as well as the cooling at ambient temperature, which was performed by means of a not forced process. Furthermore, the effect of a second annealing process was not considered. The heating maximum heating temperature was set to 450 °C as magnesium burns when treatments at higher temperature were performed. This treatment was aimed at promoting the diffusion of the protective alumina film and therefore its tribological and mechanical resistance characteristics. The effect of different temperatures applied during thermal post-treatment was assessed by investigating the morphology and microstructure of the samples, as well as by mechanical and tribological tests. The results of the characterization tests show how the protection grade that the coatings were able to provide on magnesium alloy was related to the temperature of the thermal post-treatment.

## 2. Materials and Methods

### 2.1. Materials

Samples of magnesium alloy AZ91, for which composition is reported in Table 1, were cut in squared specimens of dimension 25 × 25 mm^2^ and thickness of 3 mm. 

Before the alumina deposition with the fluidized bed technique, a lapping process was performed on the samples with an abrasive paper up to 2500 grit. Uncoated samples used as benchmark were polished to highlights the AZ91 alloy features.

**Figure 1:**
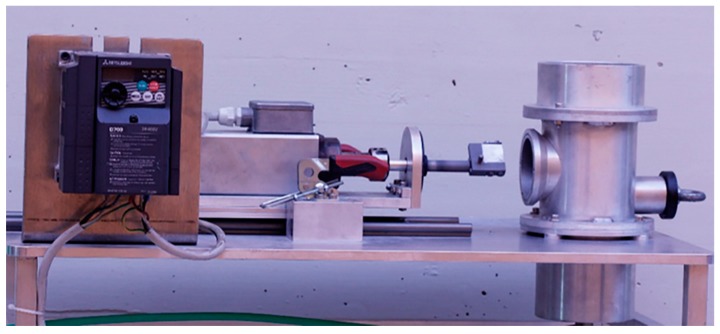
FB device exploited for the Al_2_O_3_ coating on magnesium substrate.

The samples exploited for the thermal process were coated with alumina alpha featuring a 16 mesh and a shape factor of 0.67 (Smyris Abrasivi srl, Pero, Italy) and dimensions between 1.2 and 1.4 mm, the same as previously reported [21]. Alpha phase alumina is the strongest and stiffest of the oxide ceramics. Its high hardness, excellent dielectric properties, refractoriness, good thermal properties and chemical and thermal stability make it the material of choice for a wide range of applications [27]. Indeed, its application for functional coating lead to high wear and corrosion resistance in addition to thermal and electrical insulation [28]. The coating process was performed with a rotating frequency of 6 Hz for 240 min while the fluidizing gas (air) was at a pressure of 5 bar. The fluidized bed employed for the coating process is the same exploited in [17], where the detail concerning the FB device are reported. The FB device is represented in Figure 1.

The deposition process allowed the deposition of an alumina layer of about 4 μm, measured by means of SEM images. The thermal treatments were performed with a convection oven Nabertheram P330 (Lilienthal, Germany) for 60 min. The temperatures considered for the experimental tests were 150, 300, and 450 °C. Before the thermal treatment of the samples, the oven was pre-heated to the treatment temperature.

### 2.2. Characterization

The samples produced were analysed by means of several techniques in order to evaluate the morphology of the surface in addition to the tribological and mechanical features. The characterization was performed on the coated samples before and after the thermal process and on the uncoated AZ91 alloy.

Preliminarily, the surface of the samples was investigated by optical microscopy using a stereoscope (SMZ745T, Nikon, Düsseldorf, Germany). At a later stage, the evaluation of the superficial features was exploited with a contact gauge surface profiler (Talysurf CLI 2000, Taylor-Hobson, Leicester, UK). Three-dimensional surface profiles were acquired on areas of 4 × 4 mm^2^ with a spacing and resolution of 2 μm and a measurement speed of 2 mm/s. The evaluation of the samples roughness was obtained acquiring 101 profiles of 10 mm length with a resolution of 1 μm, a spacing of 100 μm and a measurement speed of 1 mm/s. Finally, the composition and surface morphology of the deposited alumina film was examined in detail using Scanning Electron Microscopy/Energy Dispersive X-Ray Spectroscopy microscopy (SEM-EDS, FEG-SEM Leo Supra 35, Zeiss, Oberkochen, Germany). In particular, images of the surface and of the cross-section of the samples were acquired. Furthermore, an X-ray Diffraction (XRD) analysis was performed by Philips X’Pert Pro diffractometer (Amsterdam, The Netherlands), equipped with a plane mono-chromator using Cu Kα radiation (λ = 1.5418 Å). This analysis was carried out to evaluate the possible creation of new chemical species that may influence the behaviour of the samples [26].

The mechanical characterization consisted of Vickers micro-hardness tests performed with a Micro Combi tester produced by CSM Instruments (Micro Indenter MHT, CSM Instruments, Needham, MA, USA). Two different loads, 0.5 and 1 N, were considered and applied on coatings and substrate for 65 s. For each sample, 10 tests were performed to calculate the average hardness. To compare the results of the samples thermally treated with the uncoated magnesium alloy, untreated samples were subjected to the same thermal treatments as for the samples coated with alumina particles.

The tribological measurements performed were the dry-sliding linear reciprocating and the scratch test. The dry sliding linear reciprocating test, executed with a standard tribometer produced by CSM Instruments (Needham, MA, USA), was performed at a sliding speed of 8 cm/s. The counterpart was a 100Cr6 ball 6 mm in diameter with an applied load of 1 N. The wear volume was evaluated after several sliding distances, and particularly after 2, 4, 8, 20, 40, 80, 120, 240, and 500 m. The wear trails were 3D mapped with a spacing on the x and y axis respectively of 2 and 5 μm with a measurement speed of 2 mm/s. The scratch test was performed with a scratch tester CSM Instruments (Micro Indenter MHT, Needham, MA, USA), a Rockwell C diamond tip and a load up to 10 N. The measurement speed was 1 mm/min and the sliding distance 4 mm.

## 3. Results and Discussion

**Figure 2:**
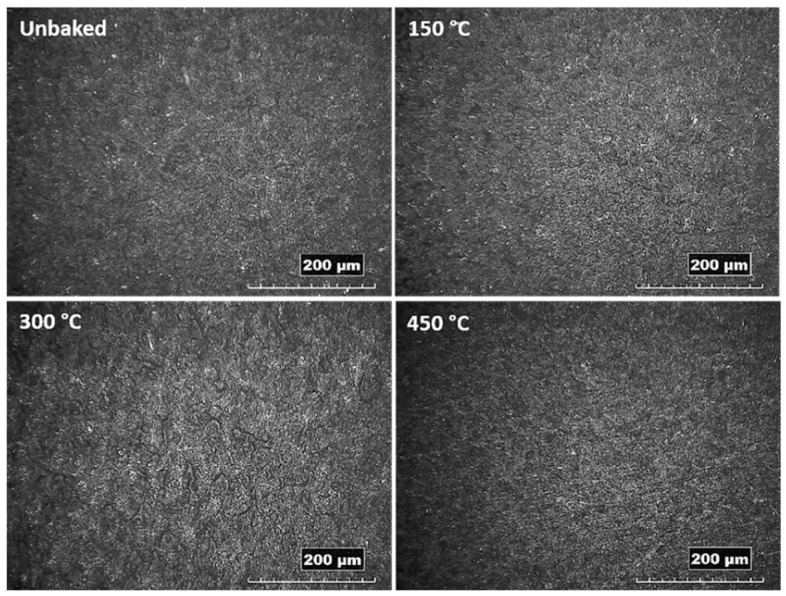
Images of the coated and heat-treated samples acquired by stereoscope.

From the images obtained by stereo microscopy and reported in Figure 2, is evident a homogeneous deposition of alumina for all the samples treated with the FB deposition process. The sample heated at 300 °C features a corrugated surface suggesting an influence of the thermal treatment on the surface morphology.

**Figure 3:**
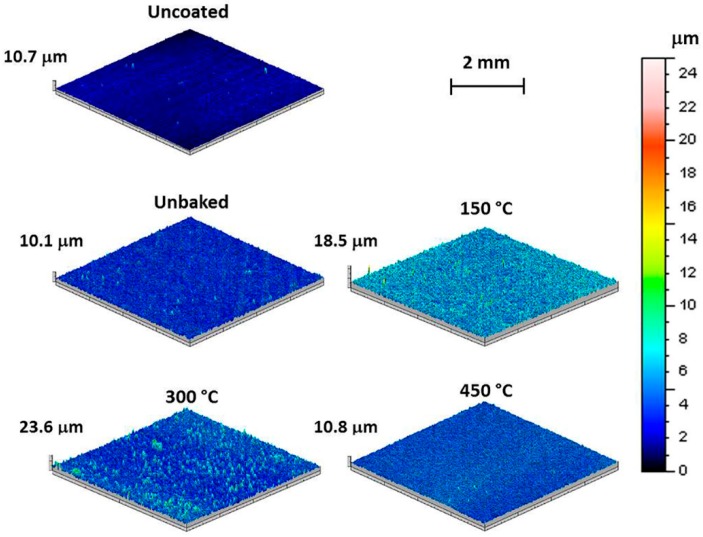
3D maps of the samples uncoated, unbaked and coated and baked at 150 °C, 300 °C, and 400 °C.

The 3D maps of the samples, proposed in Figure 3, show how the unbaked sample has a surface morphology similar to the uncoated sample as both are characterized by the peak-peak height “S_t_” of about 10 μm. Furthermore, 3D maps highlight how the morphology of the coating is a function of the treatment temperature. The sample heated to 150 °C shows an oscillation of the surface profile with a high frequency. This oscillation is reduced with the treatment temperature. Indeed, the sample treated at 300 °C assumes a profile with fewer peaks and the sample heated to 450 °C highlights a smooth surface with a morphology comparable with the uncoated sample. The S_t_ values reach the maximum value in correspondence of a heating temperature of 300 °C. These variations are due to the heat treatments exclusively, as preliminary tests demonstrated that the samples shown comparable superficial morphologies in consequence of the deposition process only.

**Figure 4:**
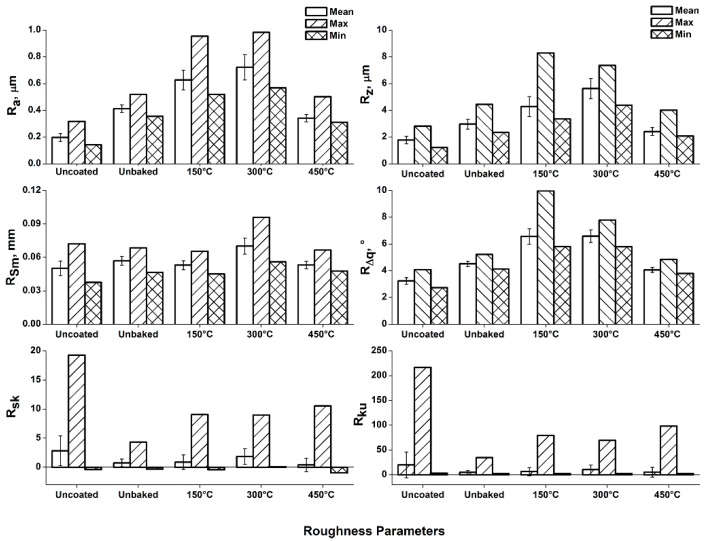
Roughness analysis of the samples surface.

The same is confirmed by the roughness analysis reported in Figure 4, which lead to the evaluation of the average roughness “R_a_”, maximum height “R_z_”, spacing “R_sm_”, and RMS (root mean square) slope “R_Δq_”. 

From the analysis of the roughness parameters it is evident how uncoated, unbaked and baked at 450 °C samples show similar values, as determined by means of 3D maps and stereoscope investigation. Compared to the unbaked sample, increasing values of R_a_, R_z_, and R_sm_ are observed for temperatures of 150 and 300 °C. Instead, there is a substantial settlement of the values of R_Δq_. The “R_sm_” increase confirms the increment of the profile oscillation frequency observed in the 3D maps. Furthermore, it can be noted that the growth of the “R_z_” value kept a constant “R_Δq_”. A trend of this type suggests the loss of surface material due to the thermal treatments undergone. In particular, magnesium has a coefficient of thermal expansion greater than alumina. Therefore, the heating treatment induces a tensile stress state on the alumina particles embedded on the surface causing a gradual detaching. With the 300 °C heating treatment, the highest value of R_sm_ was found. At this temperature there is the greatest difference in expansion between bulk and coating and, therefore, the detachment of larger alumina fragments. The heating treatment that was performed at 450 °C, about 3/4 of the magnesium melting temperature, involved a reduction in the mechanical properties of the bulk. Due to the poor mechanical properties of the bulk, the greater thermal expansion of magnesium cannot induce a stress on the alumina coating. Therefore, the detachment of coating fragments does not occur.

**Figure 5:**
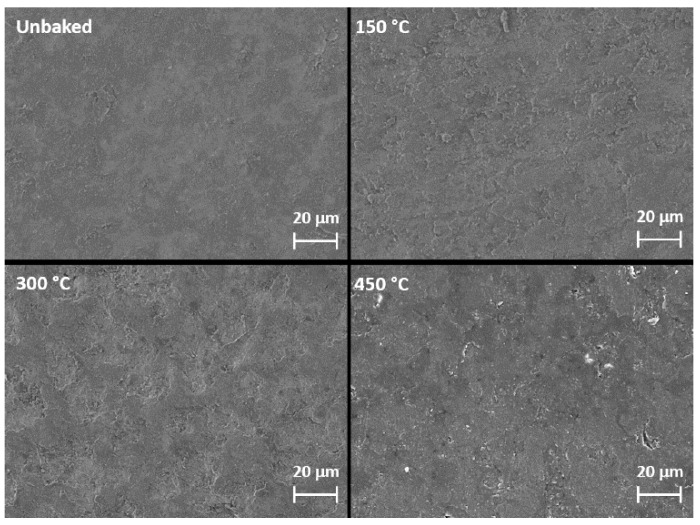
SEM images of the samples surface.

**Figure 6:**
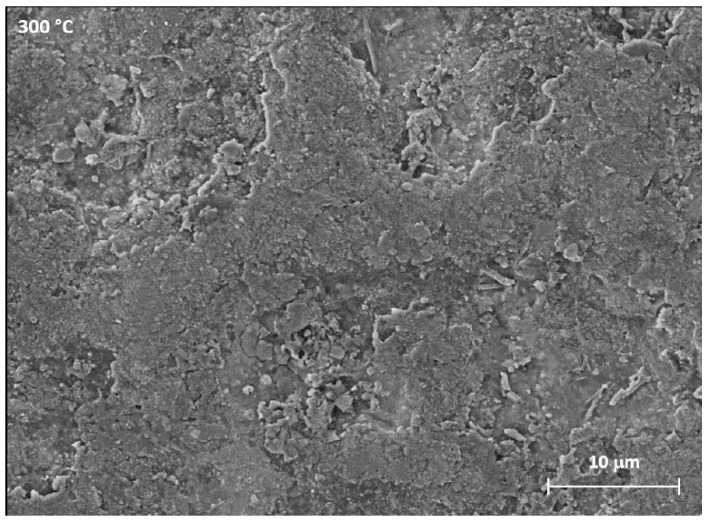
Highlight of the samples heated at 300 °C.

The SEM images of the surface of the samples shown in Figure 5 highlight the variations in morphology as a function of the heat treatment. With a greater magnification of the SEM images concerning the sample treated at 300 °C, shown in Figure 6, it is possible to observe the cracks caused by the tension state induced by the heating. 

**Figure 7:**
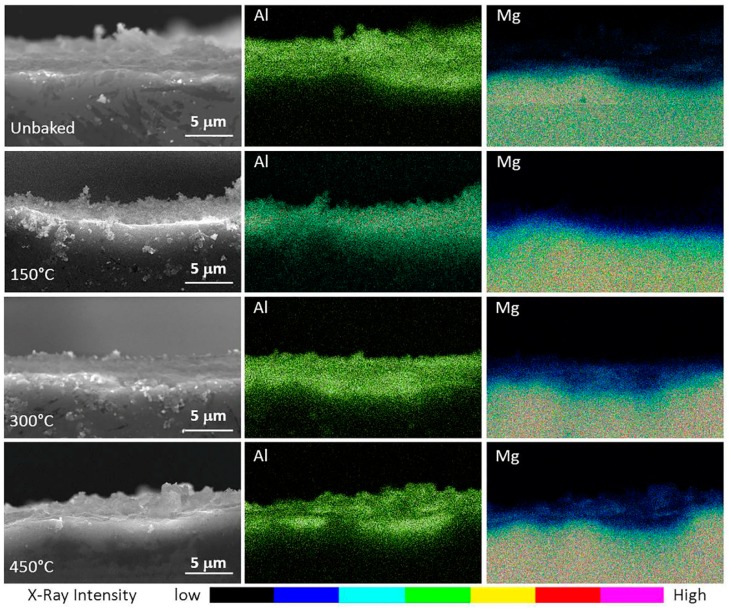
SEM-EDS images of the specimens highlighting the alumina and magnesium content of the coating.

These fractures are characterized by equiaxial geometries. The cross-section of the specimens was then analysed by means of the SEM-EDS analysis reported in Figure 7. This allowed to highlight the composition and thickness of the layer as a result of treatments at different temperatures. 

All samples had an alumina layer of about 4 µm. It can also be noted that, besides an alumina sedimentation highlighted in the previous analysis, there was a gradual diffusion of magnesium towards the superficial layers of the sample. This diffusion increases with the temperature of the thermal process and creates an interface between bulk and coating without a clear distinction. As a consequence of this interface, a greater adhesion of the coating is expected and, therefore, a better tribological behaviour. 

**Figure 8:**
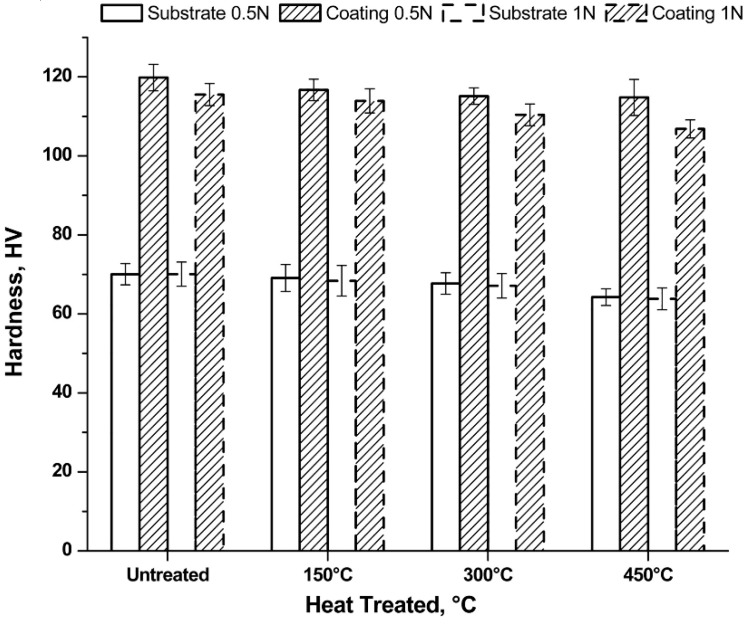
Hardness test results as function of load and heating treatment.

The mechanical tests were then performed by means of the Vickers micro-indentation, whose values are shown in Figure 8. 

Considering the same heat treatment, the uncoated samples show the same hardness values regardless of the applied load. With increasing treatment temperature, slightly decreasing hardness values are observed. This behaviour is caused by the enlargement of the grain, as well as by the reduction of the residual stress.

The alumina coating leads to an increase in hardness compared to the uncoated sample, regardless of the heat treatment suffered. The Vickers micro-hardness passes from values of about 60 HV to values of about 120 HV. Tests carried out with a minor load presented the highest hardness values, if the same treatment conditions are considered. The variation in hardness is due to the greater penetration of the indenter which, therefore, is more heavily affected by the properties of the bulk. Indeed, it is noted that the hardness reduction with increasing heating temperature is more marked for a load of 1 N with respect to the load of 0.5 N.

**Figure 9:**
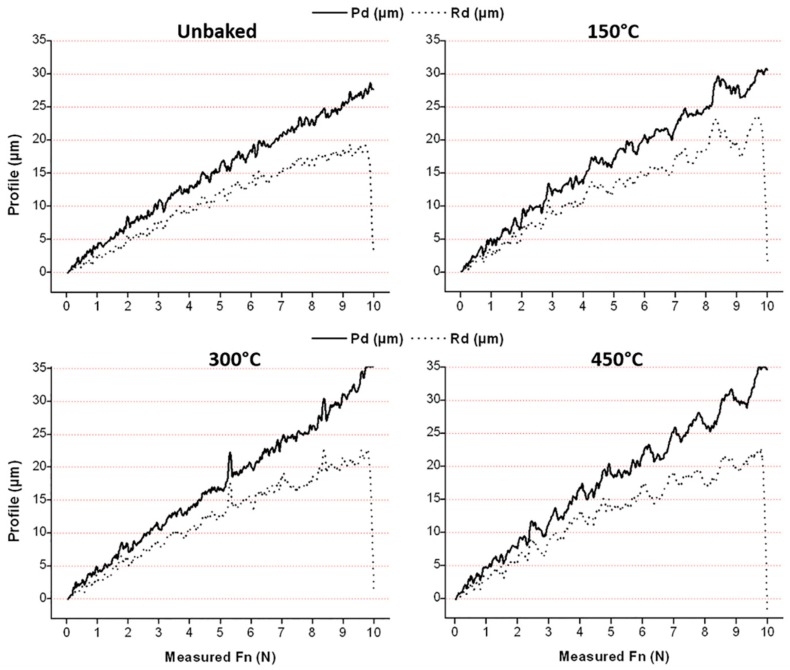
Penetration depth and residual depth of the scratch test.

**Figure 10:**
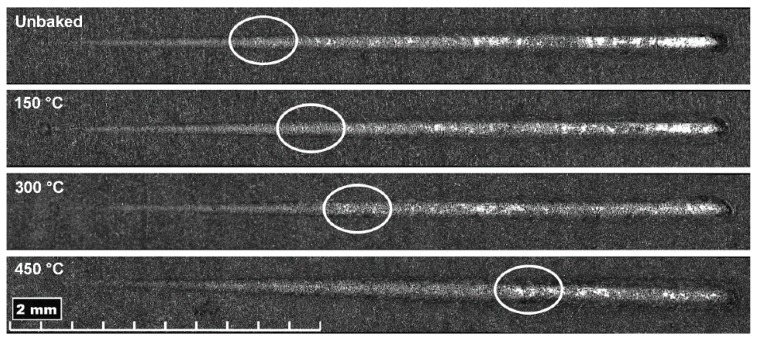
Scratch test trails with the coating detachment highlighted in the white circles.

The adhesion of the film was tested by means of a scratch test. Aim of the test was the examination of the images of the trails as well as the measurement of the penetration depth (Pd) and residual depth (Rd). The results are shown in Figure 9 and Figure 10. 

With increasing treatment temperatures, greater penetration depths are reached due to the greater ductility of the substrate, especially for the last two temperatures investigated. Residual depth is similar for all samples and, therefore, there is an increasing difference between Pd and Rd. This behaviour indicates a lower tendency of the coating to detach. Furthermore, there is a sudden drop of the residual depth at the end of the trace that increases with the temperature. This behaviour is attributable to the phenomenon of front pile up and denotes an accumulation of the coating at the end of the track and a lower tendency to fragile break. From the pictures of the scratch test trails, shown in Figure 10, it can be seen that the delamination is lower for heat treatments at higher temperatures. The damages due to the passage of the indentation are shown to increase in load as the treatment temperature increases. In fact, the damages due to the indenter begin to be evident at higher loads with the increase of the treatment temperature, as highlighted in Figure 10. The LC1 (load critical) load for the unbaked sample and the samples treated at 150, 300 and 450 °C is, respectively, of 3.9, 4.1, 5, and 7 N. Furthermore, the uncovered areas decrease with the treatment temperature.

**Figure 11:**
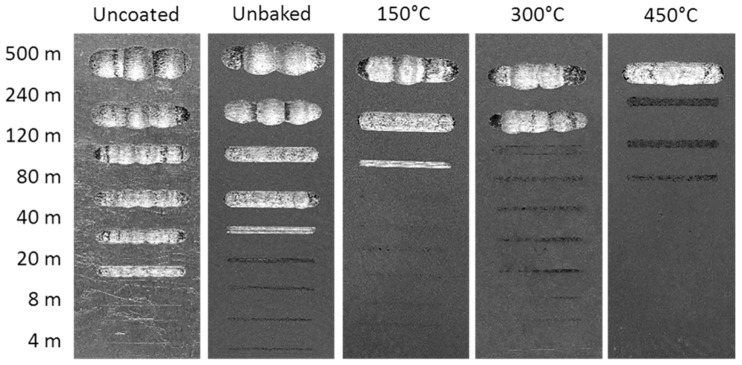
Images of the linear reciprocating trails.

**Figure 12:**
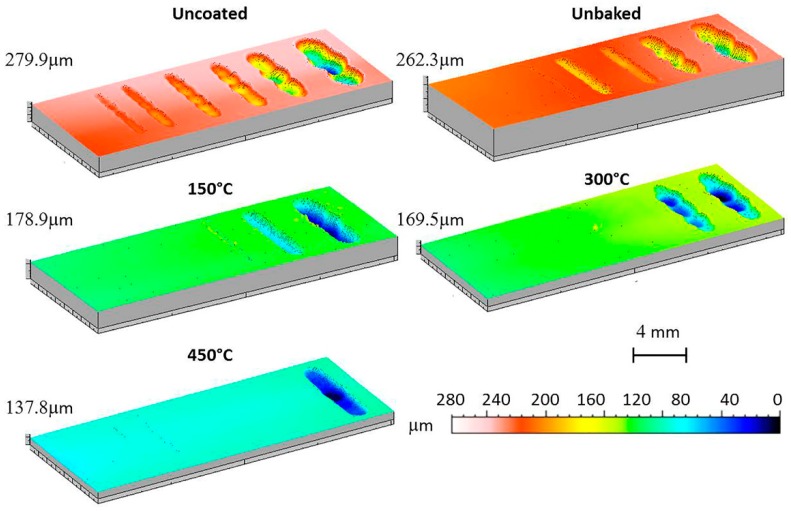
3D maps of the linear reciprocating trails.

Finally, the wear behaviour was considered by means of a dry-sliding linear reciprocating test. To evaluate the test result, the samples were photographed and the trails 3D mapped as shown in Figure 11 and Figure 12. 

**Figure 13:**
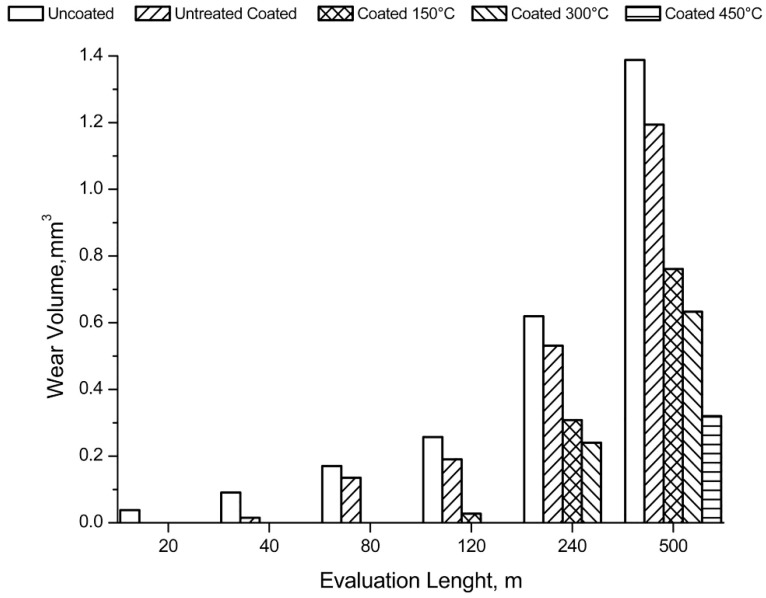
Wear volume removed during the test, as function of distance.

The volume of material removed during the test is shown in Figure 13 as a function of the sliding distance. From the pictures of the samples it is clear that the coating improves the wear behaviour of the sample. 

The first signs of wear are evident after sliding distances increase with the treatment temperature. At 150 °C the first signs appear after 120 m, while at 300 and 450 °C there are signs of wear for distances of 240 and 500 m. Furthermore, on the heat-treated samples there were evident deposits of the ball on the coating to highlight the good adhesion of the alumina powder on the substrate. The wear volume increased linearly with the sliding distance as expected. At equal distances, the volume decreases with the treatment temperature. After 500 m of sliding the wear volumes of the uncoated samples, unbaked samples, and the samples heated at 150, 300, and 450 °C were, respectively, 1.4, 1.2, 0.7, 0.6, and 0.3 mm^3^. 

Considering the 3D maps and the images, an irregular bottom is evident for the traces produced by the greater sliding distances. At the inversion point of the counterpart motion there was an increase in the friction coefficients and a phenomenon of adhesion with the substrate. This caused the dragging of the material in the wear track. Its progressive accumulation is responsible for the irregular bottom, implying the counterpart bouncing. Furthermore, in the wear patterns left on the sample before the bottom becomes irregular and with respect to the uncoated sample, there are signs parallel to the sliding axis. This wear morphology is typical of three-body wear.

**Figure 14:**
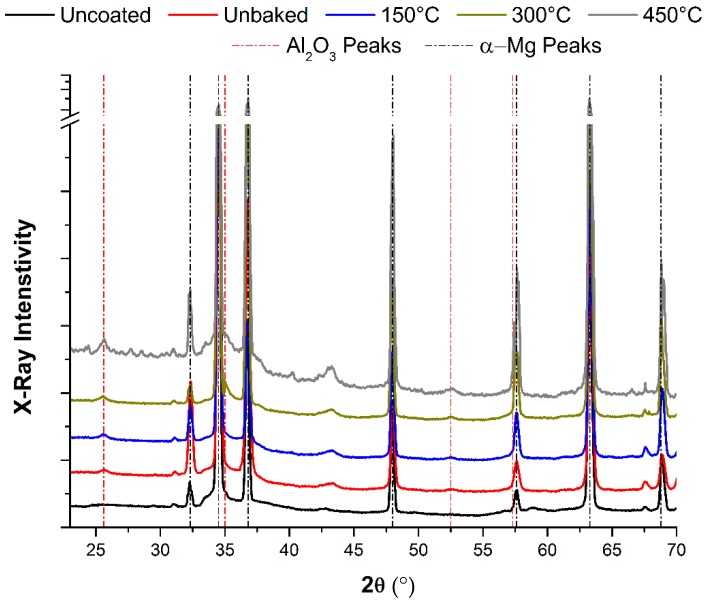
XRD analysis of the magnesium samples after the heating treatment.

The best behaviour found in thermally treated samples with respect to the uncoated and unbaked samples was considered to be due to exclusively the diffusion phenomena previously highlighted. Indeed, the XRD analysis shown in Figure 14 shows how heat treatments do not promote the formation of new chemical species capable of justifying the improved behaviour of the coated magnesium samples. The peak in correspondence of the 2ϴ value equal to 43°, which it is not present in the uncoated samples, it was due to the formation of highly crystalline periclase MgO [29]. Its formation is due to the interaction with the oxygen present in Al_2_O_3_ and rapidly develop when treated with high temperature in a non-controlled atmosphere.

Furthermore, with the heating at 450 °C there was a further improvement compared to the samples treated at lower temperatures. This temperature allows the formation of a smooth coating. The absence of crests on the surface improves the tribological behaviour of the film coated, as they are a place of concentration of tensions that may promote the breaking of the coating

## 4. Conclusions

In the work conducted, depositions of Al_2_O_3_ powder were carried out on AZ91 magnesium substrates using fluidized-bed technology. The coated samples were then heat treated at different temperatures in order to improve the performance of the coating. The various samples obtained as a result of the different heat treatments were characterized by different points of view in order to evaluate their morphology, composition, and mechanical performance. 

Through the fluidized-bed deposition process it was possible to obtain a homogeneous coating with a thickness of around 4 μm. However, surface morphology is affected by the temperature of the heat treatment. The heat treatments up to 300 °C induce stress on the alumina particles embedded on the surface, because of the different expansion coefficient that characterizes bulk and coating. This causes alumina fragments detachment and an increased surface roughness. At higher temperatures the mechanical properties of magnesium are poor and, although a different thermal expansion, the bulk cannot create a stress on the coating. The result is a smooth surface whose morphology is comparable to the unbaked sample.

Along with this phenomenon, a diffusion of magnesium towards the alumina layer which increases with treatment temperature was observed. The diffusion process along with the smooth surface are solely responsible for the improvements observed in adhesion and wear resistance of the produced coating. Indeed, no new chemical species, except magnesium oxide, was formed during thermal treatments, as observed by XRD analysis.

## Figures and Tables

**Table 1:** AZ91 composition.

Element	Weight%
Mg	90.17
Al	9.00
Zn	0.70
Mn	0.13
